# Optimizing Depth-of-Discharge in Li-Rich Halide All-Solid-State Batteries for Enhanced Capacity and Cycling Stability

**DOI:** 10.3390/ma19071409

**Published:** 2026-04-01

**Authors:** Yunan Zhou, Naibo Zhao, Xin Chen, Meiling Fan, Yang Wu, Jingchao Liu, Zhen Wu, Xiangxin Guo

**Affiliations:** 1 College of Materials Science and Engineering, Qingdao University, Qingdao 266071, China; 2 Zhejiang Green Intelligent Transportation Technology Innovation Co., Ltd., Ningbo 315300, Chinameiling.fan@geely.com (M.F.);; 3 Zhejiang Automotive Engineering Institute, Zhejiang Geely Holding Group Co., Ltd., Hangzhou 310052, China; 4 Ronggu New Material Technology (Shaoxing) Co., Ltd., Shaoxing 312000, China

**Keywords:** Li-rich layered oxides, all-solid-state batteries, halide solid electrolyte, depth-of-discharge, structural degradation, interfacial stability, voltage decay

## Abstract

**Highlights:**

**What are the main findings?**

- Optimizing discharge cut-off at 2.6 V unlocks 281.6 mAh g^−1^ capacity in HSE-LLO-ASSLBs.
- A 2.6 V cut-off activates stable ~2.85 V redox, compensating for high-voltage capacity loss.
- Excessive DOD (<2.6 V) triggers TM reduction, spinel formation, and lattice distortion.

**What are the implications of the main findings?**

- The 300-cycle capacity retention improves from 71.8% to 86.1% without coating or doping.
- Protocol-level DOD control outperforms material engineering in balancing capacity/stability.

**Abstract:**

Although halide solid electrolytes (HSEs) demonstrate a higher voltage window and superior interfacial stability toward Li-rich layered oxides (LLOs) compared to sulfide systems, HSE-based all-solid-state lithium batteries (HSE-ASSLBs) still face a fundamental trade-off between achieving high capacity and maintaining cycling stability. To resolve this issue, a rational adjustment of the depth-of-discharge (DOD) via discharge cut-off voltage control is proposed. Analysis of dQ/dV profiles and post-cycled electrodes indicates that excessive DOD (lower cut-off voltages) aggravates structural degradation and interfacial side reactions, whereas insufficient DOD (higher cut-off voltage) fails to fully utilize the compensatory capacity from low-voltage redox couples. Notably, an optimized cut-off voltage of 2.6 V activates a stable low-voltage redox reaction centered around 2.85 V, which effectively offsets high-voltage capacity loss while suppressing unfavorable interfacial evolution. As a result, the ASSLB configured with a Li_1.2_Ni_0.13_Mn_0.54_Co_0.13_O_2_ cathode and a Li_2.75_In_0.75_Zr_0.25_Cl_6_ HSE delivers an initial discharge capacity of 281.6 mAh g^−1^ at 1C and achieves significantly improved capacity retention from 71.8% to 86.1% over 300 cycles. This study confirms that DOD regulation offers a simple and effective electrochemical protocol for enabling durable high-capacity output in LLO-based ASSLBs.

## 1. Introduction

The pursuit of carbon neutrality is driving the urgent development of high-energy-density storage systems, among which lithium-ion batteries (LIBs) play a leading role [1,2]. However, the energy density of state-of-the-art LIBs is fundamentally limited by the specific capacity of commercial intercalation cathodes (e.g., ~160–200 mAh g^−1^ for LiFePO_4_ and NMC811) [3,4,5]. By contrast, Li-rich layered oxides (LLOs), generally formulated as xLi_2_MnO_3_·(1−x)LiTMO_2_ (TM = Mn, Ni, Co) [6], represent a paradigm shift. By leveraging reversible lattice-oxygen redox, LLOs can deliver exceptional capacities exceeding 250 mAh g^−1^ and cathode-level energy densities > 900 Wh kg^−1^ [7,8,9]. This unique capability makes LLOs key enablers for next-generation LIBs targeting cell-level energy densities surpassing 500 Wh kg^−1^.

However, the practical deployment of LLOs is hampered by severe electrochemical degradation induced by irreversible anionic redox, manifesting as O_2_ evolution, transition metal (TM) migration, and concomitant phase transformations [6,10,11,12]. These intrinsic issues are severely exacerbated in conventional liquid electrolytes, where continuous parasitic reactions at the cathode-electrolyte interface accelerate performance decay [13]. In response, ASSLBs have emerged as a disruptive platform, promising to physically and chemically decouple the high-voltage cathode from electrolyte decomposition.

Initial research has focused on integrating LLOs with high-conductivity sulfide solid electrolytes (SSEs) [14]. Through sophisticated interfacial engineering—exemplified by strategies like Ru doping/surface sulfidation [15] and thioglycolic acid modification [16] —SSE-based ASSLBs have demonstrated improved Li^+^ diffusion kinetics and mitigated interfacial degradation. For instance, Wu et al. reported a single-crystal LLO-based ASSLB (LLO-ASSLB) using Li_6_PS_5_Cl that delivered 316 mAh g^−1^ at 0.05C and retained 86.2% capacity after 300 cycles at 1C under 60 °C (2.0–4.8 V, 4 mg cm^−2^ areal loading) [17]. Despite these achievements, the intrinsic thermodynamic instability [18] of SSEs against LLOs’ high-voltage operation results in a persistent and resistive interface, presenting a fundamental barrier to concurrently achieving high capacity, rate capability, and long-term cycling stability.

HSEs demonstrate enhanced oxidative stability, typically >4 V [19,20]. Building on this compatibility, Sun et al. deployed dual-halide Li_3_InCl_4_._8_F_1_._2_ in a Li_2_SO_3_-coated LLO-ASSLB, achieving 248 mAh g^−1^ (areal capacity: 1.1 mAh cm^−2^) with 81.2% retention after 300 cycles at room temperature [21]. Yu et al. then enhanced interfacial transport by constructing a Li_3_PO_4_-infused Li_3_InCl_6_ electrolyte, which enabled a high capacity of 230.7 mAh g^−1^ at 0.1 C and sustained over 60% capacity retention after 431 cycles [22]. Sun et al. reported that the lifetime could be significantly extended through the integration of a dual-halide/sulfide membrane with B-doped LLOs. The resulting cell sustained 231 mAh g^−1^ at 0.1C (2.2–4.7 V) and retained 80.4% of its capacity over 2000 cycles [13]. Notwithstanding these promising results, the fundamental mechanisms governing the trade-off between specific capacity and cycling stability in HSE-LLO-ASSLBs remain inadequately understood. Furthermore, effective strategies to decouple this trade-off are highly sought after but rarely reported.

Herein, HSE-LLO-ASSLBs are constructed using a Li_1.2_Ni_0.13_Mn_0.54_Co_0.13_O_2_ cathode and a Li_2.75_In_0.75_Zr_0.25_Cl_6_ HSE. This specific LLO composition was selected as a representative model system that exhibits the characteristic high capacity and degradation patterns of Li-rich layered oxides [7], enabling a focused investigation into DOD-dependent performance trade-offs. The assembled cells with ~13.75 mg cm^−2^ areal loading delivered remarkable specific capacities of 302.9 mAh g^−1^ at 0.15C and ≥274.1 mAh g^−1^ at 1C. However, a fundamental trade-off between rate capability utilization and long-term cycling stability was revealed. To resolve this conflict, systematic control of the DOD through adjustment of the discharge cut-off voltage was implemented. As detailed in the following sections, strategic optimization of the low-voltage discharge protocol successfully decoupled this inherent trade-off. Post-cycling structural characterization reveals that this rational DOD management effectively suppresses bulk structural degradation while maintaining interfacial stability. This rational DOD management enables a final discharge capacity of 242.9 mAh g^−1^ at a deeper DOD (2.6 V cut-off) after 300 cycles, compared to 196.8 mAh g^−1^ at a shallower DOD (2.8 V cut-off), improving the capacity retention from 71.8% to 86.1%. To the best of our knowledge, no previous work has applied a shallow-depth protocol (<2.8 V) to halide-based ASSLBs; herein, simply lowering the cut-off from 2.8 V to 2.6 V boosts 300-cycle retention by 14.3% without any coating or doping, representing a paradigm shift from material- to protocol-level design.

## 2. Materials and Methods

### 2.1. Synthesis of Materials

The Li_1.2_Ni_0.13_Mn_0.54_Co_0.13_O_2_ (LLO), Li_5.5_PS_4.5_Cl_1.5_ (LPSC) and Li_2.75_In_0.75_Zr_0.25_Cl_6_ (LIZC) materials were used as-received from Huzhou Yaoning Solid-state Battery Research Institute Co., Ltd. (Huzhou, China). Prior to cell assembly, all materials were stored and handled in an Ar-filled glovebox (H_2_O & O_2_ < 0.1 ppm) and vacuum-dried at 120 °C for 12 h to remove residual moisture.

### 2.2. Materials Characterization

Scanning electron microscopy (SEM, Zeiss GeminiSEM 500, Oberkochen, Germany) was used to characterize the particle size and morphologies of the LLOs. The specific surface area and pore structure were analyzed by N_2_ adsorption–desorption measurements using the Brunauer–Emmett–Teller (BET) method (Micromeritics ASAP 2020, Norcross, GA, USA). The samples were degassed at 200 °C for 6 h under vacuum to remove adsorbed moisture and contaminants prior to measurement. Their specific compositions were determined by an inductively coupled plasma source mass spectrometer (ICP-MS, NexION 350D, PerkinElmer, Waltham, MA, USA). X-ray photoelectron spectroscopy (XPS, Thermo Fisher ESCALAB Xi+, Waltham, MA, USA) was used to probe the electronic structures of TM and oxygen ions. The acquired XPS spectra were processed with a Shirley-type background, followed by peak fitting using CasaXPS (version 2.3) software. During fitting, the full width at half maximum (FWHM) for each component was constrained within a reasonable range. Raman spectroscopy (HORIBA Scientific, Kyoto, Japan) with a 532 nm laser excitation source and a 150 μm confocal pinhole was used to analyze the phase structure. X-ray diffraction (XRD, D/MAX-2400, Tokyo, Japan; 45 kV, 40 mA) was performed over a 2θ range of 10° to 120° at a scanning rate of 1° min^−1^. The Rietveld refinements based on the XRD data were carried out using the *GSAS II* software (version 5455). TEM (JEOL JEM-2100F, Tokyo, Japan) was used to characterize the crystal lattice.

### 2.3. Electrochemical Measurements

A bilayer electrolyte configuration was adopted to leverage the respective advantages of LPSC and LIZC: the LPSC layer ensures stable contact with the Li-In anode, while the LIZC layer provides superior oxidative stability against the high-voltage LLO cathode. The ASSLBs were assembled in an Ar-filled glove box using a custom-designed mold comprising two stainless steel rods and a 10 mm-diameter ceramic sleeve. Approximately 65 mg of LPSC powder was first pressed at 125 MPa for 1 min to form a dense separator layer. Subsequently, about 30 mg of LIZC powder was stacked onto the LPSC pellet and pressed under the same conditions. The composite cathode was prepared by mixing 60 wt% LLO active material, 3 wt% VGCF, and 37 wt% LIZC, followed by ball-milling under vacuum at 320 rpm for 1 h in a planetary mill (MIXER). About 18 mg of the resulting cathode mixture was uniformly spread onto the LIZC layer and uniaxially pressed at 375 MPa for 3 min, achieving an active mass loading of ~13.75 mg cm^−2^. To improve anode interfacial stability, a bilayer of In foil (100 μm) and Li foil (50 μm) was attached to the LPSC side. Estimated thickness after 375 MPa pressing: LIZC 65 µm, LPSC 120 µm, composite cathode 70 µm (calculated from mass & pellet density); total bilayer HSE ≈ 185 µm. The assembled cell was secured in a stainless-steel casing under a 125 MPa stack pressure. Galvanostatic charge–discharge tests were performed using a LAND system at 60 °C.

## 3. Results and Discussions

### 3.1. Material Characterization of the Pristine LLOs

**Figure 1:**
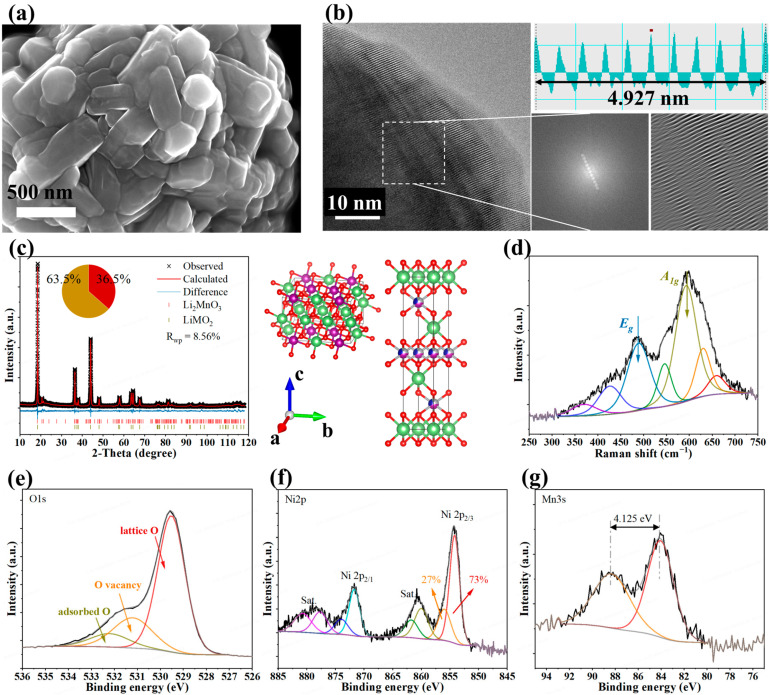
Morphology and structure of pristine LLO. (**a**) SEM images of pristine LLO samples. (**b**) TEM images, the corresponding FFT, and reverse FFT patterns of pristine LLO samples. (**c**) XRD patterns with Rietveld fits for pristine LLO samples. (**d**) Raman spectrum, and high-resolution XPS spectra of (**e**) O 1s, (**f**) Ni 2p, (**g**) Mn 3s of LLO samples.

The ionic conductivity of LIZC and LPSC was examined to 1.5 and 4.7 mS cm^−1^ at 303 K (Figure S1). The as-received LLO powder was first characterized to confirm its morphology, structure, and surface chemistry. SEM images (Figure 1a) show that the material consists of well-faceted primary particles (200–500 nm) agglomerated into dense secondary spheres. This dense secondary-sphere morphology reduces electrode porosity, enhances particle-to-particle contact, and improves Li^+^ percolation. This dense morphology is corroborated by a low specific surface area of 1.187 m^2^ g^−1^, as determined by BET measurements, which is advantageous for achieving high electrode density. The HRTEM (Figure 1b) and Fast Fourier Transform (FFT) analyses unambiguously identified lattice fringes with spacings of ~0.493 nm, corresponding to the (003) planes of a layered structure (R –3 m space group), confirming high crystallinity at the nanoscale [23,24,25].

XRD and Raman spectroscopy were employed to analyze the crystal structure. The XRD Rietveld refinement results are displayed in Table S1. The XRD pattern (Figure 1c) exhibits the characteristic signature of a well-ordered α-NaFeO_2_-type layered framework, evidenced by a high c/a ratio of 4.9948 and clear splitting of the (006)/(102) and (018)/(110) peaks [26,27,28,29]. Superlattice reflections between 20–25° confirm the presence of the Li_2_MnO_3_-type C2/m phase, which is essential for activating the anionic redox responsible for the high-capacity LLOs [30]. Rietveld refinement quantified this two-phase mixture, revealing a C2/m phase content of 36.5% and a low Li/Ni mixing degree of ~3%, both indicative of a structure conducive to Li^+^ transport and stability [31,32].

Raman spectroscopy (Figure 1d) independently verified this phase composition. The dominant peaks at ~475 cm^−1^ (*Eg*, O–M–O bending) and ~590 cm^−1^ (*A*_1*g*_, M–O stretching) are characteristic of the *R*–*3 m* phase, while weaker signals at ~330 and 420 cm^−1^ originate from the *C2/m* component [33]. Additionally, minor peaks around 625 and 650 cm^−1^ were identified and ascribed to local spinel-type environments, specifically the M–O stretching vibration of Li_x_Mn_2_O_4_ (0 < x < 1, *Fd–3m*) and the Mn–O stretching vibrations in spinel Li_4_Mn_5_O_12_, respectively [34,35].

ICP-MS confirmed the bulk composition as Li_1.1838_Ni_0.1346_Mn_0.5437_Co_0.1379_O_2_ (Table S2), showing a slight Li deficiency compared to the typical stoichiometry Li_1.2_Ni_0.13_Mn_0.54_Co_0.13_O_2_. Figure S2a displays the XPS survey spectrum, showing characteristic signals including Li 1s, C 1s, O 1s, Mn 3s, Mn 2p, Co 2p, and Ni 2p. The O 1s spectrum (Figure 1e) was fitted with the lattice oxygen (M–O) peak at 529.4 eV and a surface species component (e.g., carbonates, hydroxyls) at 531.5 eV [36]. The Ni 2p_3_/_2_ spectrum (Figure 1f) revealed a dominant Ni^2+^ contribution (73%), indicating a low average oxidation state of Ni [37]. The Mn 2p_3_/_2_ spectrum (Figure S2b) evidenced a mixed valence environment, deconvoluted into Mn^4+^ and Mn^3+^ components. This finding was corroborated by the Mn 3s spectrum (Figure 1g), where a 4.125 eV peak splitting confirmed Mn^4+^ as the dominant bulk species [38]. The Co 2p_3_/_2_ peak (Figure S2c) was fitted primarily with a satellite peak, Co^3+^, and a minor Co^2+^ component, affirming Co’s primary trivalent state, which helps stabilize the layered structure [39,40]. This multi-technique characterization confirms that the LLO material possesses the desired properties for subsequent electrochemical evaluation.

### 3.2. Electrochemical Performance and DOD Optimization

**Figure 2:**
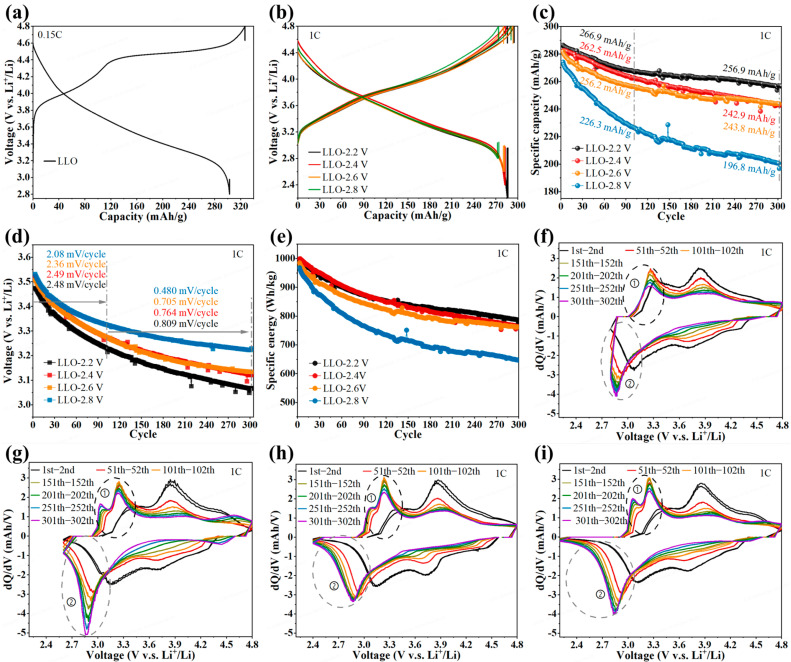
Electrochemical performance and optimization of the DOD in HSE-LLO-ASSLBs. (**a**) Initial charge–discharge voltage profile measured at 0.15 C (voltage range: 2.8–4.8 V). (**b**) First-cycle voltage profiles at 1 C under different discharge cut-off voltages (2.8 V, 2.6 V, 2.4 V, and 2.2 V). (**c**) Cycling performance evaluated at 1 C. (**d**) Average discharge voltage during cycling. (**e**) Discharge specific energy over cycles. Evolution of differential capacity (dQ/dV) profiles for cells with discharge cut-off voltages of (**f**) 2.8 V, (**g**) 2.6 V, (**h**) 2.4 V, and (**i**) 2.2 V.

The electrochemical performance of the HSE-LLO-ASSLBs was evaluated under four different discharge cut-off voltages (2.8 V, 2.6 V, 2.4 V, and 2.2 V), corresponding to progressively deeper DOD. As shown in Figure 2a,b, the cell delivered an initial discharge capacity of 302.9 mAh g^−1^ at 0.15C with an average voltage of 3.63 V in the 2.8–4.8 V window. The first-cycle 1C discharge capacities were 274.1, 281.6, 282.1, and 286.4 mAh g^−1^ for the 2.8 V, 2.6 V, 2.4 V, and 2.2 V cut-offs, respectively, confirming that additional capacity could be extracted by discharging beyond 2.8 V.

Long-term cycling tests (Figure 2c) revealed a strong dependence of capacity retention on the DOD. After 100 cycles, the retained capacities were 226.3, 256.2, 262.5, and 266.9 mAh g^−1^ for the 2.8, 2.6, 2.4, and 2.2 V cut-off voltage conditions, corresponding to capacity retentions of 82.6%, 91.0%, 93.1% and 93.2%, respectively. After 300 cycles, the final capacities were 196.8, 243.8, 242.9, and 256.9 mAh g^−1^. This trend indicates that deeper DODs generally enable access to higher specific capacities, both initially and after long-term cycling. However, this pursuit of higher capacity via deeper DOD incurs a significant trade-off in voltage stability, as evidenced by the accelerating voltage fade. Specifically, the fast average discharge voltage decay rates during the first 100 cycles were 2.08, 2.36, 2.49, and 2.48 mV cycle^−1^ for the 2.8 to 2.2 V cut-offs, respectively (Figure 2d). From the 100th to the 300th cycle, the decay rates slowed but remained DOD-dependent, with values of 0.480, 0.705, 0.764, and 0.809 mV cycle^−1^. This optimization is reflected in the cycling stability of the discharge specific energy (Figure 2e), where a significant enhancement was achieved by lowering the cut-off voltage from 2.8 V to 2.6 V, while further reductions yielded diminishing returns.

The 1C cycling voltage profiles were displayed to show the voltage decay and capacity decrease at different cut-off voltages (Figure S3a–d). To gain further insight, the corresponding dQ/dV profiles were analyzed. In the high-voltage region (>3.5 V), all cells showed consistent behavior: a stable charge peak position but negatively shifted discharge peaks, both of them attenuated, indicating a depressed discharge voltage platform and degraded high-voltage capacity contribution (Figure 2f–i).

In the lower voltage region, distinct DOD-dependent behaviors emerged. In the charge dQ/dV profiles (3–3.4 V, region ①), the main peak for all cells shifted to ~3.25 V after 50 cycles, with its intensity first increasing and then gradually weakening upon further cycling, while the peak position stabilized. Notably, for cells discharged to ≤2.6 V, an additional charge dQ/dV peak near 3–3.1 V, which mainly corresponds to the Mn^3+^/Mn^4+^ redox couple [41], appeared and intensified with cycling.

In the discharge profiles (<3.2 V, region ②), all cells exhibited a cathodic peak shift from ~3.1 to 2.85 V. The cell at the 2.6 V cut-off showed sustained signal enhancement with a sharp peak and stable baseline, whereas the 2.8 V cell exhibited slowed intensity growth, indicating restricted low-voltage capacity release. At the 2.4 and 2.2 V cut-offs, the peaks broadened and flattened, with the baseline extending toward lower voltages, signifying structural instability induced by deeper DODs [42,43]. These profiles demonstrate that a discharge cut-off of ≤2.6 V can compensate for the high-voltage capacity loss; however, the voltage-fade rate increases monotonically as the cut-off is progressively lowered to 2.2 V.

The degradation was further quantified by comparing capacity and voltage fade (Figure S4a–d). After 300 cycles at the 2.8 V cut-off, the voltage retention was 92%, while the capacity retention plummeted to 71.8%. At the 2.6 V cut-off, a more balanced profile emerged with 90% voltage retention and 86.1% capacity retention. When the cut-off was lowered to 2.4 and 2.2 V, voltage decay exceeded capacity fade within the first 100 cycles, and this imbalance persisted at the 2.2 V cut-off.

Cutting-off at ≤2.6 V yields ≥20% higher discharge capacity after 300 cycles compared with 2.8 V (Figure 2c). Further lowering the cut-off accelerates the average voltage fade rate from 0.705 mV cycle^−1^ (2.6 V) to 0.809 mV cycle^−1^ (2.2 V). The voltage fade in LLOs is strongly linked to bulk structural degradation [44]. Practical full-cells rarely utilize the <2.0 V capacity due to the low power and anode degradation. With a high-capacity Si-based anode (usually 0.3–0.6 V vs. Li/Li^+^ at the end of discharge), the discharge capacity of paired LLO cathodes below approximately 2.3 V is therefore inaccessible in real cells. For balancing cyclable capacity and voltage stability, 2.6 V is chosen as the preferred cut-off.

Beyond offering a practical protocol for charge–discharge optimization, this work demonstrates competitive performance against prior material-engineering strategies [13,19,21,22,45,46,47] as summarized in Table S3. Key advantages include a high practical areal loading of 13.75 mg cm^−2^, improved specific capacity of 302.9 and 281.6 mAh g^−1^ at 0.15 and 1C, and enhanced cycling capacity stability under the elevated temperature of 60 °C.

### 3.3. Structural and Interfacial Evolution Analysis

To further assess the interfacial kinetics, EIS was performed on cells after 300 cycles at different cut-off voltages (Figure S5). The Nyquist plots show that the interfacial charge transfer resistance remains at a similar level across all DOD conditions, indicating that the LIZC electrolyte maintains good interfacial compatibility with the LLO cathode even under deep discharge.

**Figure 3:**
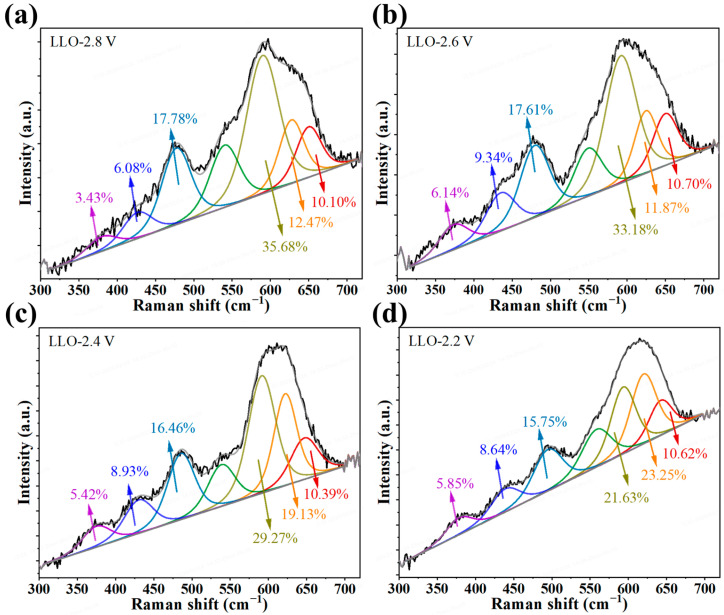
Raman spectroscopy analysis of the structural evolution in LLO cathodes after 300 cycles. (**a**–**d**) Raman spectra for LLO cycled at discharge cut-off voltages of (**a**) 2.8 V, (**b**) 2.6 V, (**c**) 2.4 V, and (**d**) 2.2 V.

Post-cycling analysis was conducted to directly correlate the DOD with the structural and interfacial evolution of the LLO cathodes. Raman spectroscopy was used to monitor the layered-to-spinel phase transition (Figure 3a–d) [48]. After 300 cycles, the spinel phase proportion remained nearly unchanged at the 2.8 and 2.6 V cut-offs. However, at the 2.4 V cut-off, the Raman bands characteristic of the layered structure (~475 and 590 cm^−1^) decreased, while the band at ~620 cm^−1^ (Li_x_Mn_2_O_4_ spinel-like phase) increased, indicating that increasing the DOD decreased the layered phase content and promoted spinel formation [49]. This trend was further exacerbated at the 2.2 V cut-off.

**Figure 4:**
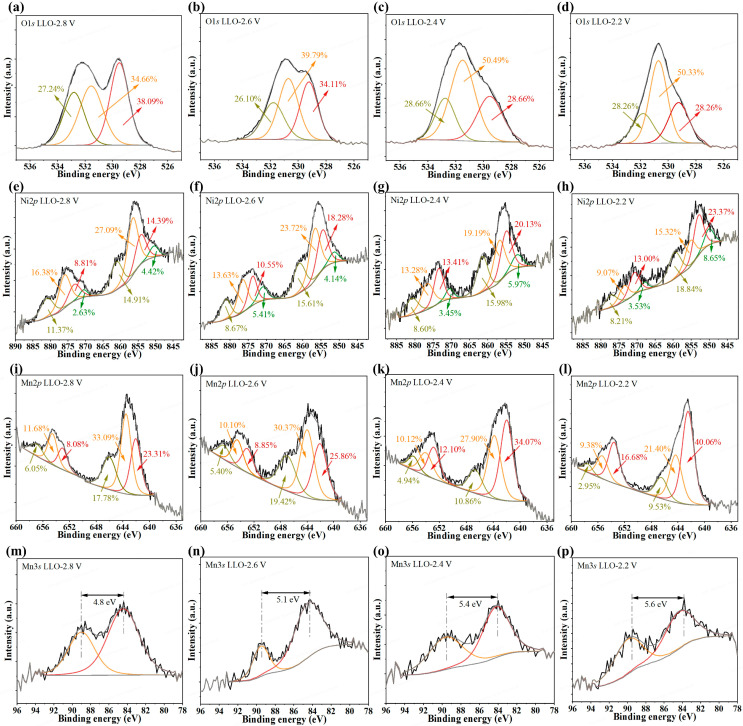
XPS analysis of the surface chemical state evolution in LLO cathodes after 300 cycles. (**a**–**d**) O 1s spectra for electrodes cycled at (**a**) 2.8 V, (**b**) 2.6 V, (**c**) 2.4 V, and (**d**) 2.2 V cut-offs. Corresponding (**e**–**h**) Ni 2p_3_/_2_, (**i**–**l**) Mn 2p_3_/_2_, and (**m**–**p**) Mn 3s spectra.

XPS analysis provided insights into the surface chemical states. The O 1s spectra (Figure 4a–d) were deconvoluted into three components: lattice oxygen (O^2−^, ~529.5 eV), oxygen vacancy (~531.5 eV), and surface species (e.g., Li_2_CO_3_, ~533.5–532.5 eV) [36,50]. After 300 cycles, the oxygen vacancy proportion slightly increased from 2.8 to 2.6 V cut-off voltage, then increased markedly at the 2.4 V and 2.2 V cut-offs, suggesting that excessive DOD promotes the formation of oxygen defects. These oxygen vacancies facilitate TM migration and promote the layered-to-spinel phase transformation, which collectively contributes to the accelerated voltage fade observed under deep discharge conditions.

The evolution of the transition metal (TM) valence states was also examined. The Ni 2p_3_/_2_ spectrum was fitted with four peaks, which are located at ~851, 853.5, 856, and 860.5 eV, respectively, attributed to the Ni^2+^ at the intermediary tetrahedral sites, Ni^2+^, Ni^3+,^ and the satellite peaks. The Ni 2p_3_/_2_ spectra (Figure 4e–h) showed that the Ni^3+^ peak areas gradually decreased while the Ni^2+^ areas increased with progressively deeper DOD (from 2.8 to 2.2 V), indicating the valence reduction of Ni. This reduction in Ni valence weakens the Ni–O bonds, destabilizes the layered framework, and reduces the reversibility of the oxygen redox activity [37,41]. Furthermore, the formation of oxygen vacancies, which is promoted under deeper discharge conditions as indicated by O 1s XPS results (Figure 4a–d), can also drive the progressive reduction in TM ions [12,36,51].

Similarly, the Mn 2p_3_/_2_ spectrum was fitted with three peaks, which are located at ~642, 643.5, and 646 eV, respectively, attributed to the Mn^3+^, Mn^4+,^ and satellite peaks. The fitted Mn 2p_3_/_2_ spectra (Figure 4i–l) revealed a decrease in the Mn^4+^ component and an increase in the Mn^3+^ component with deeper DOD. The average valence reduction of Mn was further confirmed by the Mn 3s spectra (Figure 4m–p), where the peak splitting increased from 4.8 eV (2.8 V) to 5.6 eV (2.2 V). The collective XPS results demonstrate that deeper DOD is accompanied by a more reduced state of the TMs (Ni and Mn). This reduction in TM valence is closely associated with the observed structural degradation and voltage fade.

Similarly, the Mn 2p_3_/_2_ spectra (Figure 4i–l) revealed a decrease in the Mn^4+^ component and an increase in Mn^3+^ with deeper DOD, indicating Mn valence reduction. This trend was further corroborated by the increased peak splitting in the corresponding Mn 3s spectra (Figure 4m–p). The reduction of Mn^4+^ to Mn^3+^ is coupled with the lattice oxygen evolution and the layered-to-spinel phase transformation [51]. This collective evolution underscores that deeper DOD drives bulk structural degradation, which is closely linked to the observed voltage fade. The intensification of these changes upon deeper discharge (e.g., to 2.4 V or 2.2 V) thereby accelerates structural degradation and deteriorates cycling stability.

### 3.4. Post-Cycling Microstructural Characterization

Post-cycling microstructural analysis provided direct evidence of the impact of DOD on the cathode’s structural integrity. SEM images (Figure S6a–d) show that all cycled LLO particles exhibited smoother surfaces compared to the pristine material, suggesting surface reconstruction or slight passivation during cycling. The observed fibrous VGCF conductive additive remained intact, indicating the preservation of the conductive network. FTIR spectra (Figure S7) revealed similar interfacial species across different cut-off voltages, indicating the good interfacial compatibility between LLO and the LIZC electrolyte.

**Figure 5:**
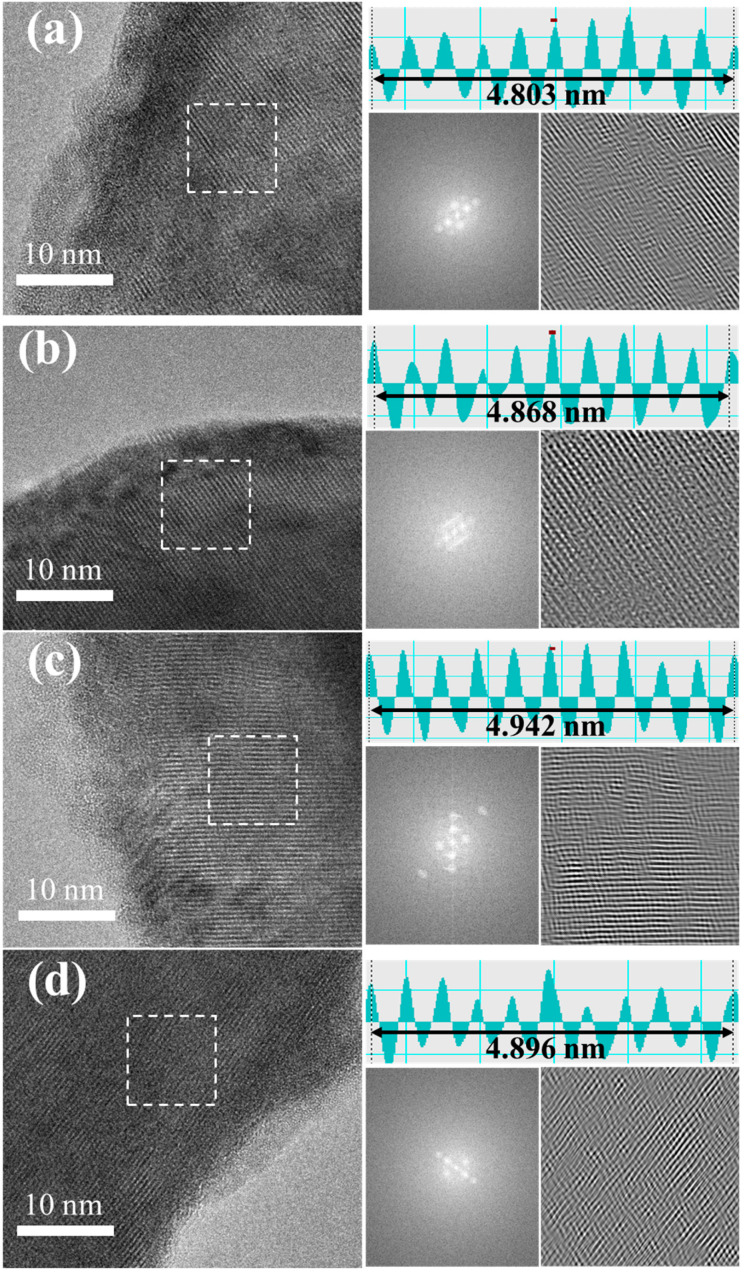
HRTEM images, FFT, and reverse FFT analysis of LLO cathodes after 300 cycles at (**a**) 2.8 V, (**b**) 2.6 V, (**c**) 2.4 V, and (**d**) 2.2 V discharge cut-off voltages.

HRTEM analysis (Figure 5a–d) revealed two critical aspects of the structural evolution. First, a very thin amorphous cathode-electrolyte interphase (CEI) layer was observed at the LLO particle edges, suggesting limited interfacial reaction and indicating good compatibility between the LLO and HSE under the tested conditions. Second, significant bulk degradation was observed in the near-surface region of the LLO particles. While the layered structure was maintained, as confirmed by the measured (003) lattice spacings of 0.480–0.495 nm, it exhibited pronounced distortions, including bending and dislocations. The severity of these lattice defects increased notably as the discharge cut-off voltage was lowered, providing a direct microstructural link between deeper DOD and accelerated bulk damage.

## 4. Conclusions

In summary, rational management of DOD through discharge cut-off voltage control is demonstrated as a facile and effective protocol for optimizing HSE-LLO-ASSLBs. A cut-off voltage of 2.6 V establishes a reasonable DOD, balancing high-capacity utilization against structural preservation. The resulting performance improvement stems not from material modification but from the structural stabilization enabled by this protocol. By avoiding excessive discharge (≤2.4 V), detrimental bulk degradation—such as accelerated spinel formation, transition-metal reduction/migration, and lattice distortion—is suppressed. This structural preservation maintains the reversible activity of the low-voltage redox couple (~2.85 V), which consistently compensates for the fading high-voltage capacity. Consequently, the cell delivers an initial capacity of 281.6 mAh·g^−1^ at 1C and retains 86.1% after 300 cycles, significantly outperforming the 71.8% retention obtained with a shallower (2.8 V) cut-off. These findings highlight that protocol-level DOD regulation provides an efficient route to decouple the capacity–stability trade-off in LLO-ASSLBs, enabling durable high-energy output without complex material engineering.

## Data Availability

The original contributions presented in this study are included in the article/Supplementary Materials. Further inquiries can be directed to the corresponding authors.

## Supplementary Materials

The following supporting information can be downloaded at: https://www.mdpi.com/article/10.3390/ma19071409/s1, Figure S1: Nyquist plots of LPSC and LIZC at 333 K; Figure S2: (a) XPS full spectrum, high-resoltion XPS spetra of (b) Mn2p and (c) Co 2p of LLO samples; Figure S3: Comparison of cycling charge and discharge profiles for cells with discharge cut-off voltages of (a) 2.8 V, (b) 2.6 V, (c) 2.4 V, and (d) 2.2 V; Figure S4: Comparison of cycling capacity and voltage retention for cells with discharge cut-off voltages of (a) 2.8 V, (b) 2.6 V, (c) 2.4 V, and (d) 2.2 V; Figure S5: Nyquist plots of HSE-LLO-ASSLBs after 300 cycles at different discharge cut-off voltages (2.8 V, 2.6 V, 2.4 V, and 2.2 V); Figure S6: SEM images of LLO cathodes after 300 cycles at discharge cut-off voltages of (a) 2.8 V, (b) 2.6 V, (c) 2.4 V, and (d) 2.2 V; Figure S7: FTIR spectra of LLOs after 300 cycles at different discharge cut-off voltages. Table S1: Crystallographic parameters for the pristine LLO from Rietveld refinement of the XRD data. Table S2: Chemical composition determined by ICP-MS. Table S3: Comparison of key parameters between this work and recent representative LLO-based ASSLBs employing halide solid electrolytes.

## References

[1] Le Y.-C., Kong W.-J., Zhao C.-Z., Shen L., Huang W.-Z., Huang Z.-Y., Hu J.-K., Zhang Q. (2026). A perspective of all-solid-state batteries with high-areal-capacity lithium-rich cathodes. J. Energy Chem..

[2] Quilty C.D., Wu D., Li W., Bock D.C., Wang L., Housel L.M., Abraham A., Takeuchi K.J., Marschilok A.C., Takeuchi E.S. (2023). Electron and Ion Transport in Lithium and Lithium-Ion Battery Negative and Positive Composite Electrodes. Chem. Rev..

[3] Ahmed S., Trask S.E., Dees D.W., Nelson P.A., Lu W., Dunlop A.R., Polzin B.J., Jansen A.N. (2018). Cost of automotive lithium-ion batteries operating at high upper cutoff voltages. J. Power Sources.

[4] Jarolimek K., Risko C. (2021). Modification of the LiFePO_4_ (010) Surface Due to Exposure to Atmospheric Gases. ACS Appl. Mater. Interfaces.

[5] You Y., Celio H., Li J., Dolocan A., Manthiram A. (2018). Modified High-Nickel Cathodes with Stable Surface Chemistry Against Ambient Air for Lithium-Ion Batteries. Angew. Chem. Int. Ed..

[6] Zeng L., Liang H., Qiu B., Shi Z., Cheng S., Shi K., Liu Q., Liu Z. (2023). Voltage Decay of Li-Rich Layered Oxides: Mechanism, Modification Strategies, and Perspectives. Adv. Funct. Mater..

[7] Liu Y., Lu Y., Jing S., Zhang Y., Kuang W., Zhang Z., Liu Y., Zhang K., Xu Z., Liu X. (2026). Artificial Anion Vacancies Tailoring Local Oxygen Structure of Li-Rich Cathode for High-Energy and Durable All-Solid-State Batteries. Energy Storage Mater..

[8] Yuan L., Peng W., Zhan Z., Wang J., Feng Y., Yan Y., Yu R., Wang C., Wang Z., Guo H. (2025). Enhancing Ionic Transport at Primary Interparticle Boundaries of Polycrystalline Lithium-Rich Oxide in All-Solid-State Batteries. Angew. Chem. Int. Ed..

[9] Shi J., Xiao D., Ge M., Yu X., Chu Y., Huang X., Zhang X., Yin Y., Yang X., Guo Y. (2018). High-Capacity Cathode Material with High Voltage for Li-Ion Batteries. Adv. Mater..

[10] Qiu S., Bai J., Wang P., Xiao K., Liu Y., Wang S., Zhu X., Xiao Y., Zhao B., Sun Y. (2026). In Situ Magnetism Decoupling Gradient-Regulated Mn–O Interaction Mechanism on Stabilizing Li-Rich Cathodes. Nano Lett..

[11] Jin L., Du G., Liu P., Gu T., Gao R., Abdelkader A.M., Hua W., Xu M., Peng L., Qiu B. (2026). Voltage Decay and Capacity Loss in Lithium-Rich Manganese Oxide Cathodes: Atomic Origins, Mesoscopic Heterogeneities, and Macroscopic Evolution. Adv. Mater..

[12] Zhang Y.-H., Zhang S., Hu N., Liu Y., Ma J., Han P., Hu Z., Wang X., Cui G. (2024). Oxygen vacancy chemistry in oxide cathodes. Chem. Soc. Rev..

[13] Sun S., Zhao C., Liu G., Wang S., Fu Z., Kong W., Li J., Chen X., Zhao X., Zhang Q. (2025). Boosting Anionic Redox Reactions of Li-Rich Cathodes through Lattice Oxygen and Li-Ion Kinetics Modulation in Working All-Solid-State Batteries. Adv. Mater..

[14] Schwietert T.K., Arszelewska V.A., Wang C., Yu C., Vasileiadis A., de Klerk N.J.J., Hageman J., Hupfer T., Kerkamm I., Xu Y. (2020). Clarifying the relationship between redox activity and electrochemical stability in solid electrolytes. Nat. Mater..

[15] Wang Y., Wu D., Chen P., Lu P., Wang X., Chen L., Li H., Wu F. (2024). Dual-Function Modifications for High-Stability Li-Rich Cathode Toward Sulfide All-Solid-State Batteries. Adv. Funct. Mater..

[16] Song G., Lee S., Kim T., Jung M.S., Kim K., Choi S.H., Lee S., Park J., Lee M., Park C. (2024). Mechano-Electrochemical Behavior of Nanostructured Li,- and Mn-Rich Layered Oxides with Superior Capacity Retention and Voltage Decay for Sulfide-Based All-Solid-State Batteries. Adv. Energy Mater..

[17] Wu Y., Li C., Zheng X., Zhao W., Wang H., Gu J., Cheng Y., Lin Y., Su Y., Ren F. (2024). High Energy Sulfide-Based All-Solid-State Lithium Batteries Enabled by Single-Crystal Li-Rich Cathodes. ACS Energy Lett..

[18] Ren F., Liang Z., Zhao W., Zuo W., Lin M., Wu Y., Yang X., Gong Z., Yang Y. (2023). The nature and suppression strategies of interfacial reactions in all-solid-state batteries. Energy Environ. Sci..

[19] Zhang A., Wang J., Yu R., Zhuo H., Wang C., Ren Z., Wang J. (2023). Practical Application of Li-Rich Materials in Halide All-Solid-State Batteries and Interfacial Reactions between Cathodes and Electrolytes. ACS Appl. Mater. Interfaces.

[20] Wang S., Bai Q., Nolan A.M., Liu Y., Gong S., Sun Q., Mo Y. (2019). Lithium Chlorides and Bromides as Promising Solid-State Chemistries for Fast Ion Conductors with Good Electrochemical Stability. Angew. Chem. Int. Ed..

[21] Sun S., Zhao C.-Z., Yuan H., Fu Z.-H., Chen X., Lu Y., Li Y.-F., Hu J.-K., Dong J., Huang J.-Q. (2022). Eliminating interfacial O-involving degradation in Li-rich Mn-based cathodes for all-solid-state lithium batteries. Sci. Adv..

[22] Yu R., Wang C., Duan H., Jiang M., Zhang A., Fraser A., Zuo J., Wu Y., Sun Y., Zhao Y. (2023). Manipulating Charge-Transfer Kinetics of Lithium-Rich Layered Oxide Cathodes in Halide All-Solid-State Batteries. Adv. Mater..

[23] Huang Q., Liu J., Chen X., Zhang P., Lu L., Ren D., Ouyang M., Liu X. (2025). Recent Progress and Challenges of Li-Rich Mn-Based Cathode Materials for Solid-State Lithium-Ion Batteries. Adv. Mater..

[24] Noh M., Cho J. (2012). Optimized Synthetic Conditions of LiNi_0.5_Co_0.2_Mn_0.3_O_2_Cathode Materials for High Rate Lithium Batteries via Co-Precipitation Method. J. Electrochem. Soc..

[25] Freire M., Lebedev O.I., Maignan A., Jordy C., Pralong V. (2017). Nanostructured Li_2_MnO_3_: A disordered rock salt type structure for high energy density Li ion batteries. J. Mater. Chem. A.

[26] Lu C., Yang S., Wu H., Zhang Y., Yang X., Liang T. (2016). Enhanced electrochemical performance of Li-rich Li 1.2 Mn 0.52 Co 0.08 Ni 0.2 O 2 cathode materials for Li-ion batteries by vanadium doping. Electrochim. Acta.

[27] Huang Z., Wang Z., Jing Q., Guo H., Li X., Yang Z. (2016). Investigation on the effect of Na doping on structure and Li-ion kinetics of layered LiNi0.6Co0.2Mn0.2O2 cathode material. Electrochim. Acta.

[28] Liu Y., Fan X., Zhang Z., Wu H.-H., Liu D., Dou A., Su M., Zhang Q., Chu D. (2018). Enhanced Electrochemical Performance of Li-Rich Layered Cathode Materials by Combined Cr Doping and LiAlO_2_ Coating. ACS Sustain. Chem. Eng..

[29] Li Q., Li G., Fu C., Luo D., Fan J., Li L. (2014). K^+^-Doped Li_1.2_Mn_0.54_Co_0.13_Ni_0.13_O_2_: A Novel Cathode Material with an Enhanced Cycling Stability for Lithium-Ion Batteries. ACS Appl. Mater. Interfaces.

[30] Wang E., Xiao D., Wu T., Liu X., Zhou Y., Wang B., Lin T., Zhang X., Yu H. (2022). Al/Ti Synergistic Doping Enhanced Cycle Stability of Li-Rich Layered Oxides. Adv. Funct. Mater..

[31] Luo Y.-H., Pan Q.-L., Wei H.-X., Huang Y.-D., Tang L.-B., Wang Z.-Y., He Z.-J., Yan C., Mao J., Dai K.-H. (2022). Towards Ni-rich layered oxides cathodes with low Li/Ni intermixing by mild molten-salt ion exchange for lithium-ion batteries. Nano Energy.

[32] Thackeray M.M., Kang S.-H., Johnson C.S., Vaughey J.T., Benedek R., Hackney S.A. (2007). Li2MnO3-stabilized LiMO2 (M = Mn, Ni, Co) electrodes for lithium-ion batteries. J. Mater. Chem..

[33] Hu S., Li Y., Chen Y., Peng J., Zhou T., Pang W.K., Didier C., Peterson V.K., Wang H., Li Q. (2019). Insight of a Phase Compatible Surface Coating for Long-Durable Li-Rich Layered Oxide Cathode. Adv. Energy Mater..

[34] Liu S., Liu Z., Shen X., Li W., Gao Y., Banis M.N., Li M., Chen K., Zhu L., Yu R. (2018). Surface Doping to Enhance Structural Integrity and Performance of Li-Rich Layered Oxide. Adv. Energy Mater..

[35] Zhang X., Shi J., Liang J., Yin Y., Zhang J., Yu X., Guo Y. (2018). Suppressing Surface Lattice Oxygen Release of Li-Rich Cathode Materials via Heterostructured Spinel Li_4_Mn_5_O_12_ Coating. Adv. Mater..

[36] Hou P., Li F., Zhang H., Huang H. (2020). Stabilizing the cationic/anionic redox chemistry of Li-rich layered cathodes by tuning the upper cut-off voltage for high energy-density lithium-ion batteries. J. Mater. Chem. A.

[37] Shi J.-L., Zhang J.-N., He M., Zhang X.-D., Yin Y.-X., Li H., Guo Y.-G., Gu L., Wan L.-J. (2016). Mitigating Voltage Decay of Li-Rich Cathode Material via Increasing Ni Content for Lithium-Ion Batteries. ACS Appl. Mater. Interfaces.

[38] Ding X., Luo D., Cui J., Xie H., Ren Q., Lin Z. (2020). An Ultra-Long-Life Lithium-Rich Li1.2Mn0.6Ni0.2O2 Cathode by Three-in-One Surface Modification for Lithium-Ion Batteries. Angew. Chem. Int. Ed..

[39] Deng Y.-P., Yin Z.-W., Wu Z.-G., Zhang S.-J., Fu F., Zhang T., Li J.-T., Huang L., Sun S.-G. (2017). Layered/Spinel Heterostructured and Hierarchical Micro/Nanostructured Li-Rich Cathode Materials with Enhanced Electrochemical Properties for Li-Ion Batteries. ACS Appl. Mater. Interfaces.

[40] Sun Z., Xu L., Dong C., Zhang H., Zhang M., Ma Y., Liu Y., Li Z., Zhou Y., Han Y. (2019). A facile gaseous sulfur treatment strategy for Li-rich and Ni-rich cathode materials with high cycling and rate performance. Nano Energy.

[41] Pimenta V., Sathiya M., Batuk D., Abakumov A.M., Giaume D., Cassaignon S., Larcher D., Tarascon J.-M. (2017). Synthesis of Li-Rich NMC: A Comprehensive Study. Chem. Mater..

[42] Wu Z., Cheng Y., Shi Y., Xia M., Zhang Y., Hu X., Zhou X., Chen Y., Sun J., Liu Y. (2022). Restriction of voltage decay by limiting low-voltage reduction in Li-rich oxide materials. J. Colloid Interface Sci..

[43] Nayak P.K., Grinblat J., Levi M., Levi E., Kim S., Choi J.W., Aurbach D. (2016). Al Doping for Mitigating the Capacity Fading and Voltage Decay of Layered Li and Mn-Rich Cathodes for Li-Ion Batteries. Adv. Energy Mater..

[44] Guo H., Wei Z., Jia K., Qiu B., Yin C., Meng F., Zhang Q., Gu L., Han S., Liu Y. (2019). Abundant nanoscale defects to eliminate voltage decay in Li-rich cathode materials. Energy Storage Mater..

[45] Li X., Ye Q., Wu Z., Zhang W., Huang H., Xia Y., Gan Y., He X., Xia X., Zhang J. (2023). High-voltage all-solid-state lithium batteries with Li3InCl6 electrolyte and LiNbO3 coated lithium-rich manganese oxide cathode. Electrochim. Acta.

[46] Xu X., Chu S., Xu S., Guo S., Zhou H. (2024). Self-constructing a lattice-oxygen-stabilized interface in Li-rich cathodes to enable high-energy all-solid-state batteries. Energy Environ. Sci..

[47] Kong W.-J., Zhao C.-Z., Shen L., Sun S., Huang X.-Y., Xu P., Lu Y., Huang W.-Z., Li J.-L., Huang J.-Q. (2024). Bulk/Interfacial Structure Design of Li-Rich Mn-Based Cathodes for All-Solid-State Lithium Batteries. J. Am. Chem. Soc..

[48] Nayak P.K., Grinblat J., Levi E., Levi M., Markovsky B., Aurbach D. (2017). Understanding the influence of Mg doping for the stabilization of capacity and higher discharge voltage of Li- and Mn-rich cathodes for Li-ion batteries. Phys. Chem. Chem. Phys..

[49] Wei Z., Zhang W., Wang F., Zhang Q., Qiu B., Han S., Xia Y., Zhu Y., Liu Z. (2015). Eliminating Voltage Decay of Lithium-Rich Li_1.14_Mn_0.54_Ni_0.14_Co_0.14_O_2_ Cathodes by Controlling the Electrochemical Process. Chem. A Eur. J..

[50] Foix D., Sathiya M., McCalla E., Tarascon J.-M., Gonbeau D. (2016). X-ray Photoemission Spectroscopy Study of Cationic and Anionic Redox Processes in High-Capacity Li-Ion Battery Layered-Oxide Electrodes. J. Phys. Chem. C.

[51] Hu E., Yu X., Lin R., Bi X., Lu J., Bak S., Nam K.-W., Xin H.L., Jaye C., Fischer D.A. (2018). Evolution of redox couples in Li- and Mn-rich cathode materials and mitigation of voltage fade by reducing oxygen release. Nat. Energy.

