# Supplementary material for: Optimizing Depth-of-Discharge in Li-Rich Halide All-Solid-State Batteries for Enhanced Capacity and Cycling Stability
Source: Materials (Basel). 2026 Apr 1;19(7):1409. doi: 10.3390/ma19071409 (PMC13073891; doi:10.3390/ma19071409)
Supplement: Supplementary file 1 [file materials-19-01409-s001.zip › materials-4151370-supplementary.pdf]

materials

Supplementary Materials

Optimizing Depth-of-Discharge in Li-Rich Halide All-Solid-State Batteries for Enhanced Capacity and Cycling Stability

Yunan Zhou [1,2,3,†], Naibo Zhao [2,†], Xin Chen [1,†], Meiling Fan [2], Yang Wu [2], Jingchao Liu [2], Zhen Wu [1,2,3,*] and Xiangxin Guo [1,4,*]

[1] College of Materials Science and Engineering, Qingdao University, Qingdao 266071, China
[2] Zhejiang Green Intelligent Transportation Technology Innovation Co., Ltd., Ningbo 315300, China
[3] Zhejiang Automotive Engineering Institute, Zhejiang Geely Holding Group Co., Ltd., Hangzhou 310052, China
[4] Ronggu New Material Technology (Shaoxing) Co., Ltd., Shaoxing 312000, China
* Correspondence: wuzhenmse@163.com (Z.W.); xxguo@qdu.edu.cn (X.G.)
† These authors contributed equally to this work.

Table S1. Crystallographic parameters for the pristine LLO from Rietveld refinement of the XRD data.

a (Å)	c (Å)	c/a	Z_{oxy} [a]	Ni in Li layer
2.8500	14.2352	4.9948	0.2419	3.012%

[a] Zoxy is the O position (0, 0, Z_{oxy}) at 6c sites.

**Rhombohedral Phase (*R–3m*)**						
Name	X	Y	Z	Biso	Occ	Mult
O2	0.00000	0.00000	0.24188	0.74313	0.16667	6
Li1	0.00000	0.00000	0.00000	0.53138	0.08082	3
Ni1	0.00000	0.00000	0.50000	0.36470	0.00874	3
Mn1	0.00000	0.00000	0.50000	0.36470	0.04500	3
Co1	0.00000	0.00000	0.50000	0.36470	0.01150	3
Li2	0.00000	0.00000	0.50000	0.36470	0.01525	3
Ni2	0.00000	0.00000	0.00000	0.53138	0.00251	3
Li3	0.00000	0.00000	0.00000	0.36470	0.00251	3

Table S2. Chemical composition determined by ICP-MS.

	Element	Content (wt.%)
Principal Elements	Li	9.18
	Ni	8.83
	Co	9.08
	Mn	33.37

Table S3. Comparison of key parameters between this work and recent representative LLO-based ASSLBs employing halide solid electrolytes.

Ref.	Cathode	Catholyte	Modification Strategy	Temp. (°C)	Loading (mg cm^{-2})	Voltage Window (V)	Initial Capacity (mAh g^{-1})	Cycling Capacity Performance
This work	$Li_{1.2}Ni_{0.13}Mn_{0.54}Co_{0.13}O_2$	$Li_{2.75}In_{0.75}Zr_{0.25}Cl_6$	DOD-Regulation	60	~13.75	2.6-4.8	302.9 (0.15C) 281.6 (1C)	86.1% after 300 cycles (1C)
[21]	$Li_{1.2}Ni_{0.13}Mn_{0.54}Co_{0.13}O_2$	$Li_3InCl_{4.8}F_{1.2}$	Li_2SO_3 coating	RT	~4.2	2.3-4.6	248 (0.1C)	81.2% after 300 cycles (1C)
[19]	$Li_{1.15}Ni_{0.265}Mn_{0.53}Co_{0.055}O_2$	Li_3InCl_6	$LiNbO_3$ coating	RT	~10.7	2.0-4.8	221 (0.1C)	50% after 100 cycles (1C)
[22]	/	Li_3InCl_6 (HSE)	Li_3PO_4-infused HSE	RT	/	2.0-4.8	231 (0.1C)	60% after 431 cycles (0.5C)
[45]	/	Li_3InCl_6	$LiNbO_3$ coating	RT	/	2.3-4.8	185 (0.05C)	67% after 100 cycles (0.05C)
[46]	single-crystal Li_2RuO_3	Li_3InCl_6	Stabilizing O^{n-} by In-O	60	/	2.0-4.5	294 (0.1C)	99.6% after 300 cycles (0.5C)
[47]	$Li_{1.2}Ni_{0.13}Mn_{0.54}Co_{0.13}O_2$	Li_3InCl_6	Li_2WO_4 bulk embedded structure	25	~15.3	2.2-4.6	218.6 (0.1C)	84.1% after 1200 cycles (1C)
[13]	$Li_{1.2}Ni_{0.13}Mn_{0.54}Co_{0.13}O_2$	Li_3InCl_6	B-doping	RT	/	2.2-4.7	231 (0.1C)	80.4% after 2000 cycles

https://doi.org/10.3390/xxxxx

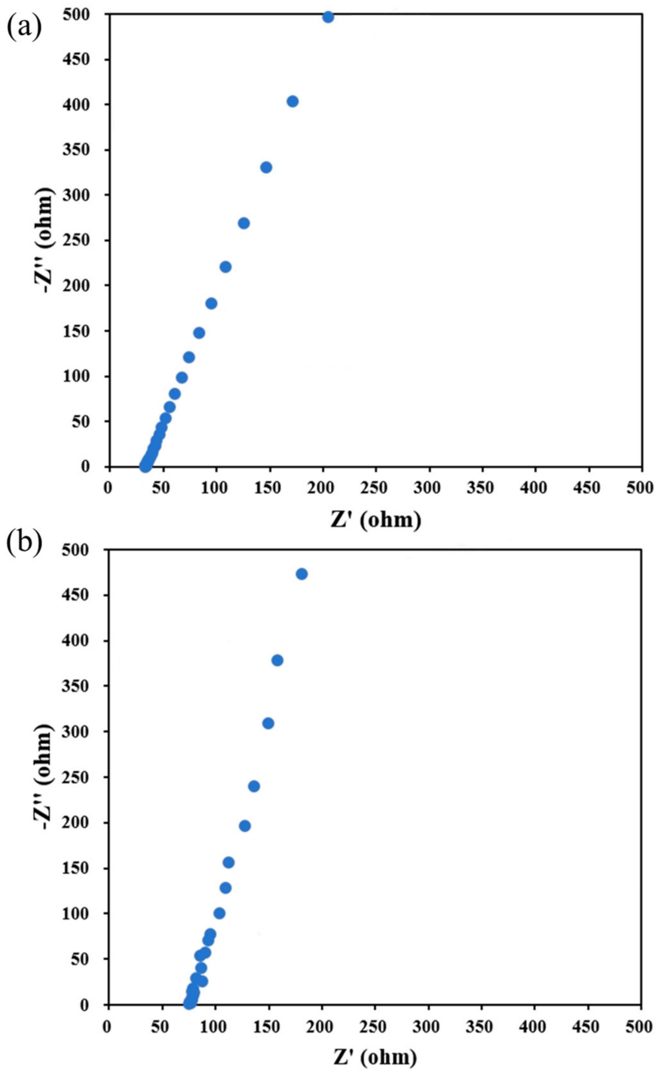

Figure S1. Nyquist plots of (a) LPSC and (b) LIZC at 333 K.

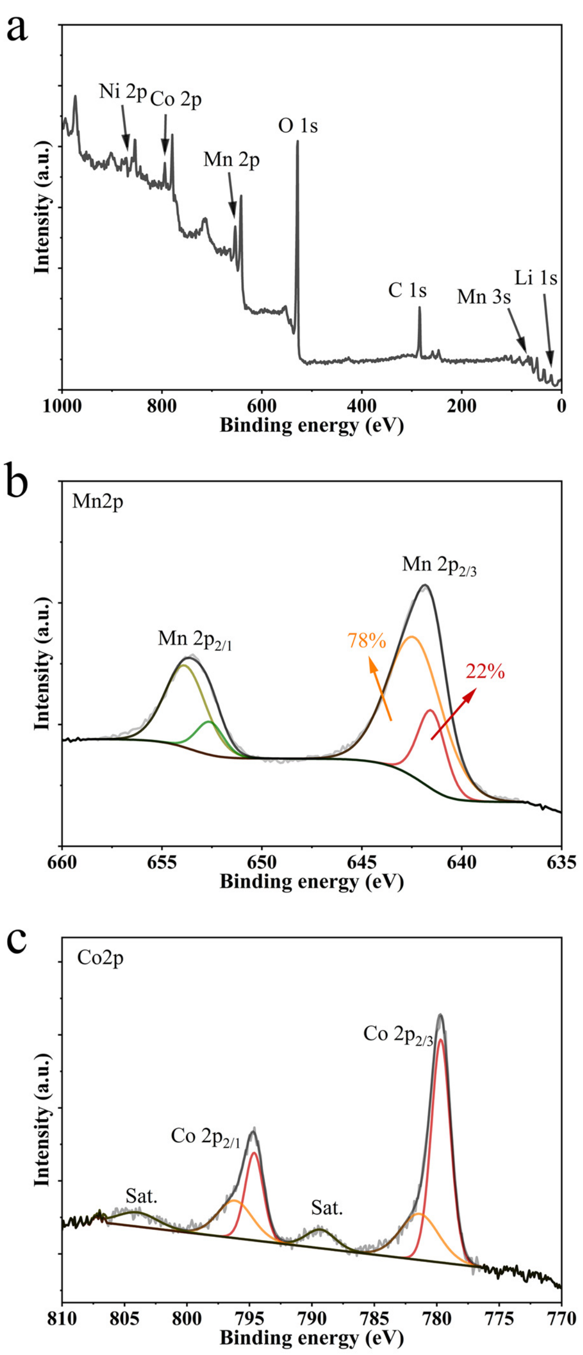

Figure S2. (a) XPS full spectrum, high-resolution XPS spetra of (b) Mn2p and (c) Co 2p of LLO samples.

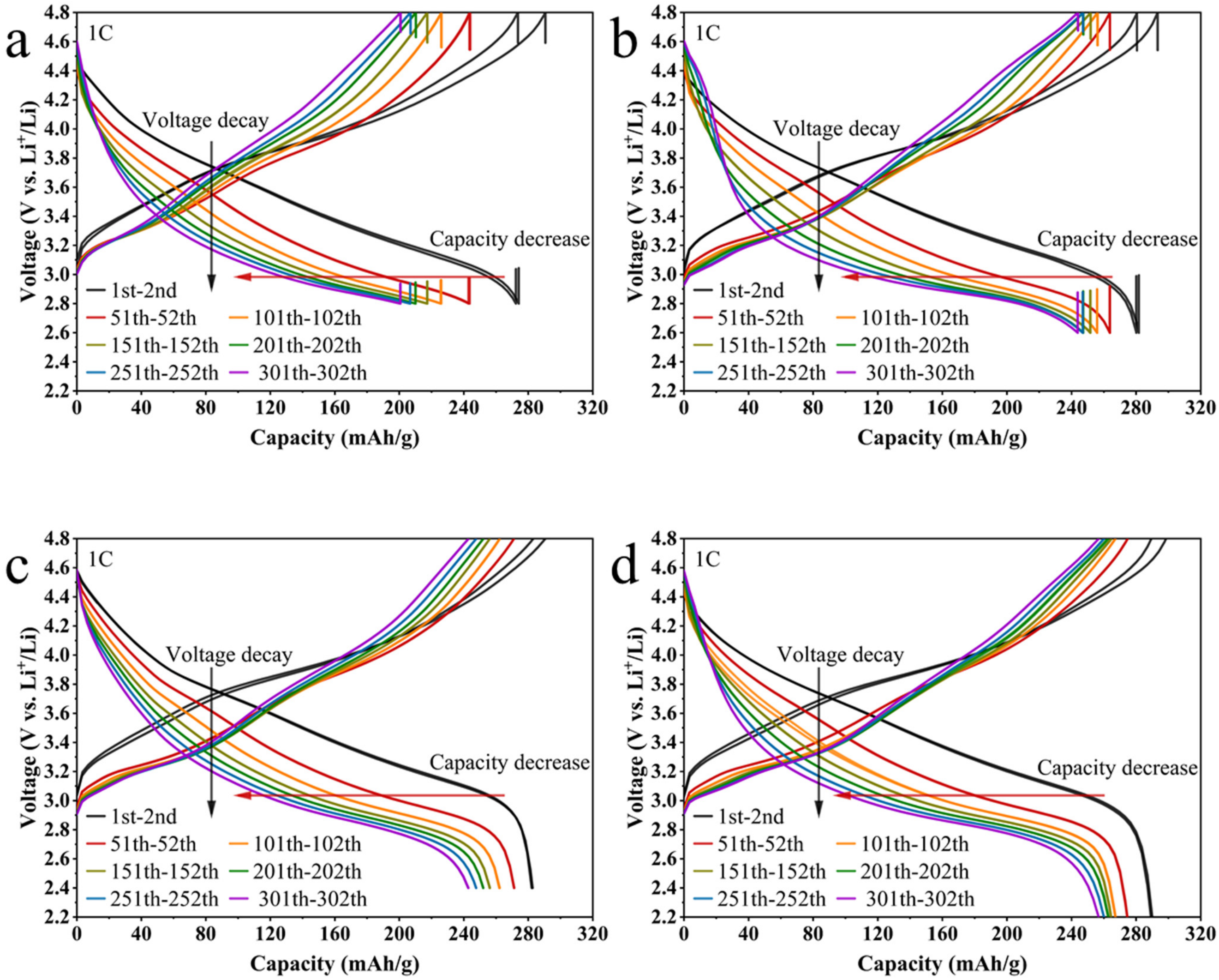

Figure S3. Comparison of cycling charge and discharge profiles for cells with discharge cut-off voltages of (a) 2.8 V, (b) 2.6 V, (c) 2.4 V, and (d) 2.2 V.

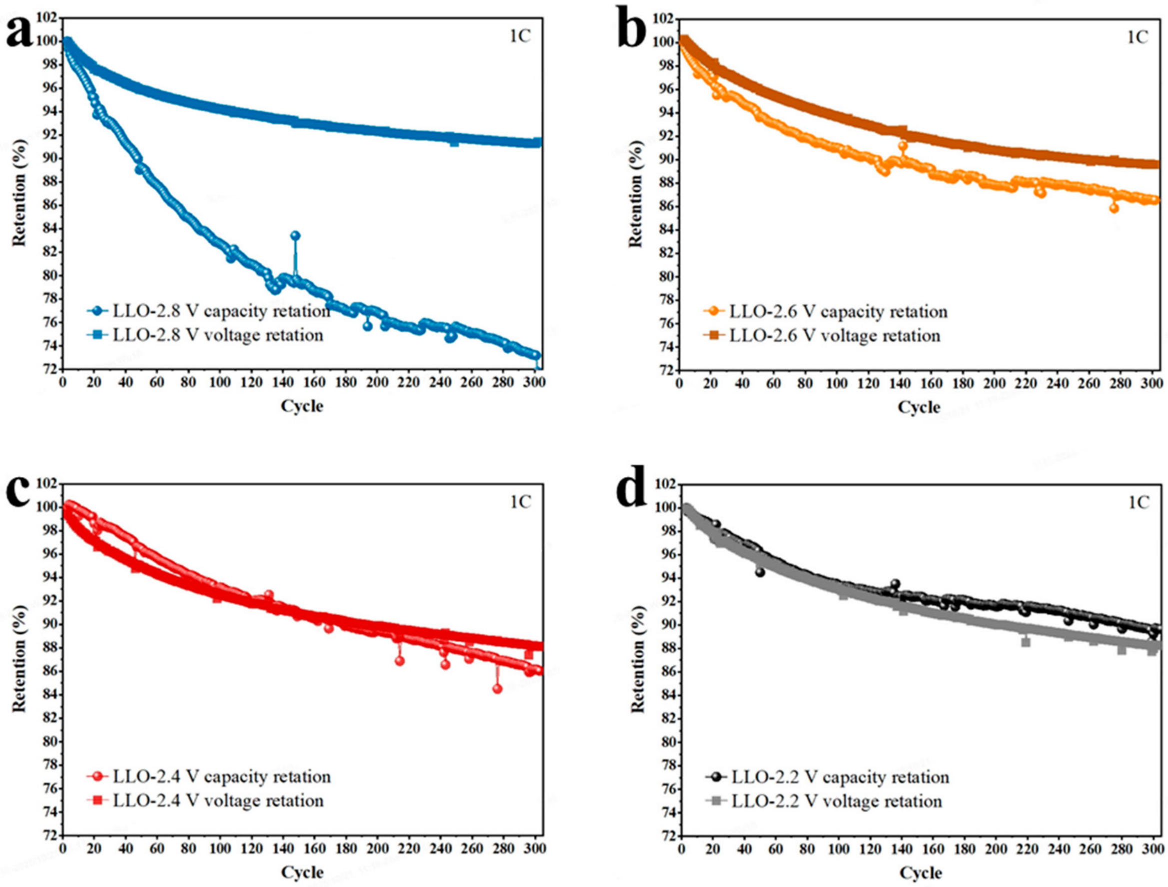

Figure S4. Comparison of cycling capacity and voltage retention for cells with discharge cut-off voltages of (a) 2.8 V, (b) 2.6 V, (c) 2.4 V, and (d) 2.2 V.

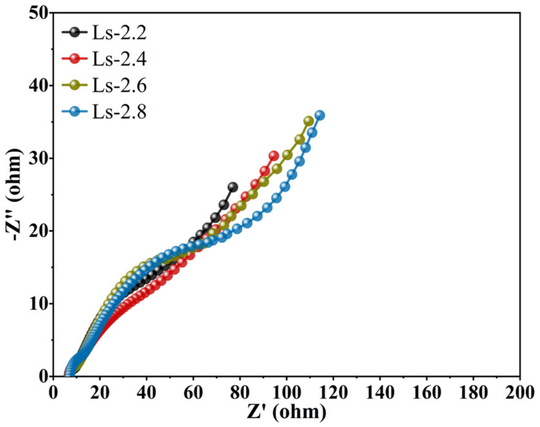

Figure S5. Nyquist plots of HSE-LLO-ASSLBs after 300 cycles at different discharge cut-off voltages (2.8 V, 2.6 V, 2.4 V, and 2.2 V).

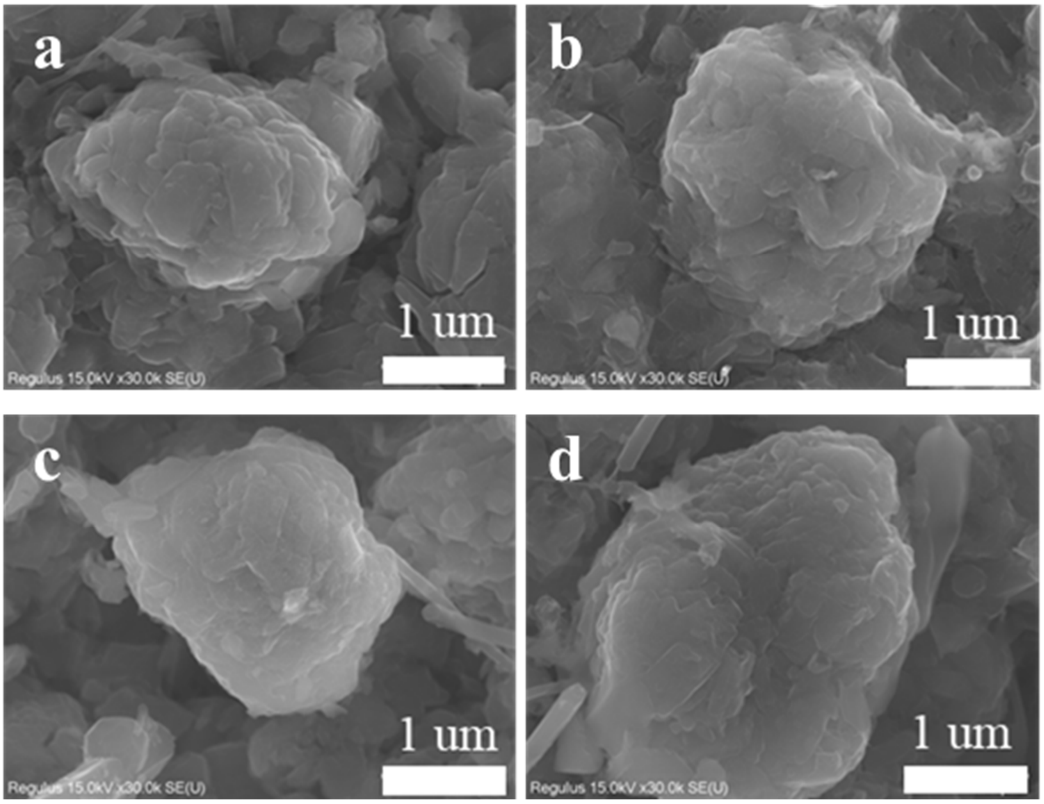

Figure S6. SEM images of LLO cathodes after 300 cycles at discharge cut-off voltages of (a) 2.8 V, (b) 2.6 V, (c) 2.4 V, and (d) 2.2 V.

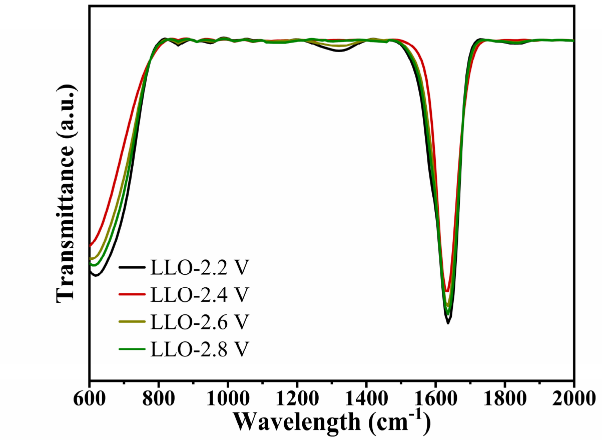

Figure S7. FTIR spectra of LLOs after 300 cycles at different discharge cut-off voltages
